# Duration of dual antiplatelet therapy in patients treated with percutaneous coronary intervention for coronary chronic total occlusion

**DOI:** 10.1371/journal.pone.0176737

**Published:** 2017-05-05

**Authors:** Seung Hwa Lee, Jeong Hoon Yang, Seung-Hyuk Choi, Taek Kyu Park, Woo Jin Jang, Young Bin Song, Joo-Yong Hahn, Jin-Ho Choi, Hyeon-Cheol Gwon

**Affiliations:** 1 Division of Cardiology, Department of Medicine, Heart, Stroke and Vascular Institute, Samsung Medical Center, Sungkyunkwan University School of Medicine, Seoul, Republic of Korea; 2 Division of Cardiology, Department of Medicine, Samsung Changwon Hospital, Sungkyunkwan University School of Medicine, Changwon, Republic of Korea; University of Messina, ITALY

## Abstract

**Background:**

The duration of dual antiplatelet therapy (DAPT) after drug-eluting stent implantation in coronary chronic total occlusion (CTO) remains unclear.

**Methods:**

We retrospectively analyzed a total of 512 patients treated with percutaneous coronary intervention (PCI) in the Samsung Medical Center CTO registry. Patients were separated into ≤ 12-month (199, 38.9%) vs. > 12 month (313, 61.1%) based on DAPT duration with aspirin and clopidogrel. The primary outcome was major adverse cardiac and cerebrovascular event (MACCE) during follow-up.

**Results:**

Median follow-up duration was 67 (interquartile range: 51–84) months. MACCE occurred in 43 patients (21.6%) in the ≤ 12-month and 55 patients (17.6%) in the > 12-month groups. In the propensity-matched population, the rate of MACCE did not differ significantly between the ≤ 12-month and > 12-month group (19.4% vs. 18.8%; hazard ratio [HR], 0.95; 95% confidential interval [CI], 0.52–1.76, p = 0.88). Moreover, moderate or severe bleeding according to BARC criteria (type 2, 3 or 5) was also similar between the ≤ 12-month and > 12-month group (2.5% vs. 1.9%; HR, 1.00; 95% CI, 0.20–4.96, p = 0.99).

**Conclusion:**

Among patients treated with PCI for CTO, DAPT with durations of ≤ 12-month showed similar long-term clinical outcomes compared to > 12-month DAPT.

## Introduction

Dual antiplatelet therapy (DAPT) with aspirin and P2Y12 receptor inhibitor is recommended for patients undergoing percutaneous coronary intervention (PCI).[1] Several former trials suggested that there is no net clinical benefit of DAPT beyond 12 months after drug-eluting stent (DES) implantation in terms of ischemic and bleeding event, although recent large randomized trials report benefits associated with DAPT over 12 months, which were accompanied by an increased number of bleeding events.[2–6] In addtion, clinical presentation which associated with unstable plaque or plaque rupture may benefit with long-duration DAPT although their prevalence is not negligible in stable patients.[7] Therefore, long-duration DAPT is difficult to apply in all patients, and multiple factors have to be integrated: type of stent, clinical presentation, type of dual antiplatelet therapy, coronary lesion complexity, and patient compliance.[8]

The optimal duration of DAPT may differ in patients with coronary chronic total occlusion (CTO) because such patients are at higher risk of ischemic events.[9] Although it has become more common to undergo PCI to treat CTO and the success rates of such treatment have increased, the optimal duration of DAPT after DES implantation in patients with CTO remains unclear.[10–13] Therefore, the aim of the present study was to compare long-term clinical outcomes between patients with CTO who were treated with prolonged DAPT and those treated for up to 12 month after PCI with DES.

## Methods

### Study population

This study was retrospective analysis with selected CTO patients from prospective registry. Between March 2003 and February 2012, 2,024 consecutive patients were enrolled in the Samsung Medical Center CTO registry. Clinical, laboratory and outcome data were collected by a trained study coordinator using a standardized case report form and protocol. If necessary, additional information was documented by contacting the principal investigators and/or by review of hospital records. The Institutional Review Board of Samsung Medical Center approved the study protocol and waived the requirement for informed consent. The inclusion criteria for the registry were: 1) patients treated for PCI with DES for CTO, 2) at least one CTO detected on diagnostic coronary angiography, and 3) symptomatic angina and/or a positive functional ischemia study. Exclusion criteria included: 1) failed PCI, 2) medical therapy alone without PCI, 3) previous coronary artery bypass graft surgery, 4) history of cardiogenic shock or cardiopulmonary resuscitation, 5) ST-segment elevation myocardial infarction (MI) during the preceding 48 hours, and 6) use of warfarin or antiplatelet agent, other than aspirin and clopidogrel. Patients with major adverse cardiac and cerebrovascular events (MACCE) during the first 12 month excluded.

### Definitions and outcomes

A CTO lesion was defined as the obstruction of a native coronary artery with thrombolysis in myocardial infarction (TIMI) flow grade 0 with an estimated duration longer than three months.[3] Duration was estimated based on the interval from the last episode of acute coronary syndrome (ACS) or in patients with no history of ACS, from the first episode of exertional angina consistent with the location of the occlusion or previous coronary angiogram. Successful PCI was defined as final residual stenosis less than 20% of the vessel diameter, with TIMI flow grade ≥ 2 after revascularization, as assessed by visual estimation on angiography.

The primary outcome was the MACCE during follow-up. The secondary outcomes were each of MACCE, cardiac death, or moderate or severe bleeding during the same period (assessed according to the Bleeding Academic Research Consortium [BARC] criteria).[14] MI was defined as recurrent symptoms with new electrocardiographic changes compatible with MI or cardiac markers at least twice the upper limit of normal.[15] Periprocedural enzyme elevation was not included in this definition of MI. The incidence of bleeding was also evaluated according to Global Utilization of Streptokinase and Tissue Plasminogen Activator for Occluded Arteries (GUSTO) and TIMI criteria.

### Statistical analysis

Comparisons for continuous variables were made using t-tests or the Wilcoxon rank-sum test when applicable, and results are presented as mean ± standard deviation or median with interquartile ranges. Differences between groups were evaluated using chi-square or Fisher’s exact tests for categorical data. We used landmark analysis in this study, and we defined landmark of DAPT discontinuation at 12 month. Survival curves were constructed using Kaplan-Meier estimates and compared with the log-rank test. Univariable and multivariable Cox proportional hazard regression models were used to compare reductions in the risks of outcomes. Variables associated with MACCE (acute coronary syndrome, location of CTO, multivessel disease and newer generation stent) were included in multiple cox-regression analyses to search for independent predictors. To reduce treatment-selection bias and potential confounding factors, we performed rigorous adjustments for the baseline and lesion characteristics of the patients using their propensity scores, which we estimated using multiple logistic-regression analyses. Model discrimination was assessed with c-statistics, and model calibration was assessed with Hosmer- Lemeshow statistics (P = 0.22). An absolute standardized difference < 10% for the measured covariate suggested an appropriate balance between the groups. The covariate balance achieved by matching was assessed by calculating the absolute standardized differences in covariates between the ≤ 12-month and > 12-month groups. An absolute standardized mean difference of <10% for the measured covariate suggests appropriate balance between groups. In the propensity score-matched population, continuous variables were compared with a paired t-test, as appropriate, and categorical variables were compared with the McNemar’s test of symmetry, as appropriate. The reduction in the risk of outcome was compared using the stratified Cox regression model. Statistical analyses were performed with SPSS 20.0.0 (IBM Corp. Armonk, NY, USA). All tests were two-tailed and p < 0.05 was considered statistically significant.

## Results

### Baseline and procedural characteristics

**Fig 1:**
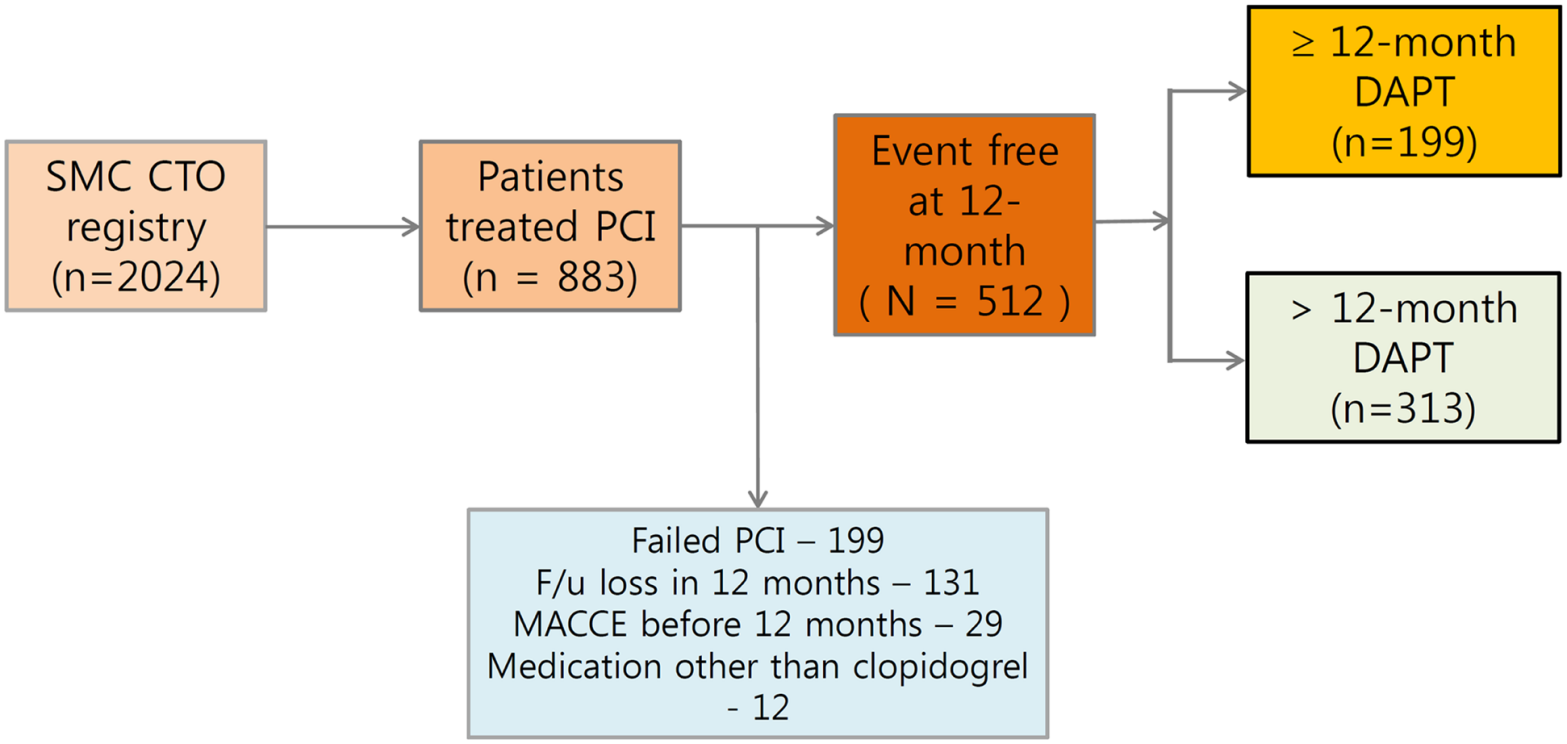
Group distribution in the registry.

The patient flow of the study is shown in Fig 1. The baseline characteristics of the CTO patients in the ≤ 12-month (N = 199), and > 12-month (N = 313) DAPT groups are described in Table 1.

**Table 1:** Baseline characteristics according to duration of dual antiplatelet therapy.

	Total population				Propensity-matched population		
Variables	≤ 12-month DAPT (N = 199)	> 12-month DAPT (N = 313)	p-value	Standardized mean difference	≤12-month DAPT (N = 185)	>12-month DAPT (N = 185)	Standardized mean difference
Age	59.2 (±10.50)	61.4 (±11.35)	0.03	-20.58	59.3 (±10.54)	59.4 (±11.3)	-0.72
Male	158 (79.4)	257 (82.1)	0.45	6.69	146 (78.9)	144 (77.8)	2.67
Medical history							
Diabetes	73 (36.7)	134 (42.8)	0.17	-12.68	67 (36.2)	73 (39.5)	-6.71
Hypertension	109 (54.8)	193 (61.7)	0.12	-13.8	100 (54.1)	101 (54.6)	-1.08
Chronic kidney disease	5 (2.5)	26 (8.3)	0.007	-36.93	5 (2.7)	5 (2.7)	0
Smoking	70 (35.2)	101 (32.3)	0.50	6.07	66 (35.7)	68 (36.8)	-2.26
Dyslipidemia	71 (35.7)	112 (35.8)	0.98	-0.22	66 (35.7)	71 (38.4)	-5.63
History of MI	32 (16.1)	61 (19.5)	0.33	9.26	30 (16.2)	29 (15.7)	1.47
History of PCI	17 (8.5)	53 (16.9)	0.007	-29.94	17 (9.2)	17 (9.2)	0
History of stroke	5 (2.5)	27 (8.6)	0.005	-38.96	5 (2.7)	4 (2.2)	3.45
Left ventricular ejection fraction	59.4 (±10.21)	58.0 (±11.07)	0.15	13.89	58.5 (±10.0)	58.7 (±10.87)	7.17
Acute coronary syndrome	48 (24.1)	79 (25.2)	0.78	-2.61	46 (24.9)	40 (21.6)	7.56
SYNTAX score	19.4 (±8.37)	20.4 (±9.03)	0.11	-11.47	19.6 (±8.49)	19.5 (±8.47)	0.41

Data are presented as mean ± standard deviation or n (%).

DAPT, dual antiplatelet therapy; MI, myocardial infarction; SYNTAX, Synergy between PCI with Taxus and Cardiac Surgery.

**Fig 2:**
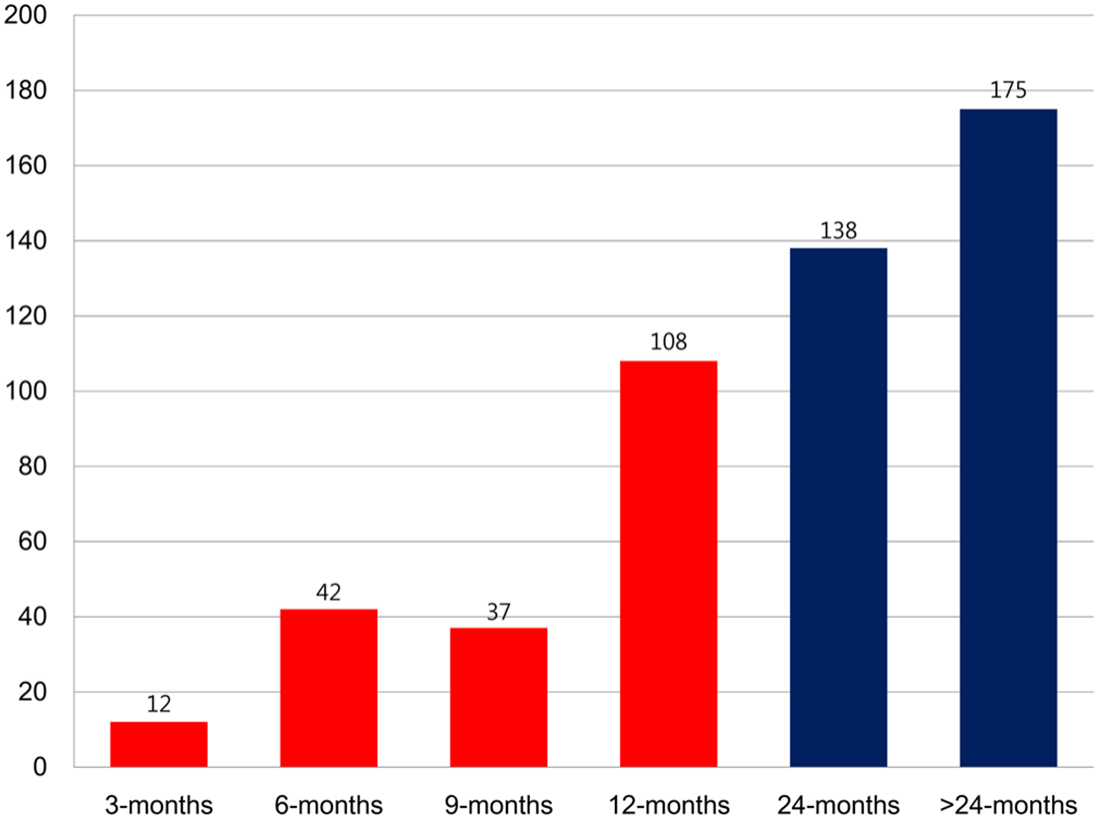
The distribution of DAPT duration.

The median duration of DAPT was 292 days (IQR, 184–363 days) in the ≤ 12-month group. Compared with patients in the > 12-month DAPT group, those in the ≤ 12-month group were younger and had a lower incidence of chronic kidney disease, history of PCI, and history of stroke. Fig 2 shows the distribution of DAPT duration.

Table 2 shows the lesion and procedural characteristics.

**Table 2:** Lesion and procedural charateristics according to duration of dual antiplatelet therapy.

	Total population				Propensity-matched population		
Variables	≤ 12-month DAPT (N = 199)	> 12-month DAPT (N = 313)	p-value	Standardized mean difference	≤12-month DAPT (N = 185)	>12-month DAPT (N = 185)	Standardized mean difference
CTO lesion							
Left anterior descending artery	90 (45.2)	147 (47.0)	0.70	-3.48	83 (44.9)	81 (43.8)	2.17
Left circumflex artery	52 (26.1)	80 (25.6)	0.89	1.3	49 (26.5)	51 (27.6)	-2.45
Right coronary artery	82 (41.2)	141 (45.0)	0.39	-7.79	78 (42.2)	78 (42.2)	0
Multivessel disease	134 (67.3)	205 (65.5)	0.67	3.92	127 (68.7)	121 (65.4)	6.9
CTO at proximal to mid	152 (76.4)	240 (76.7)	0.94	-0.69	141 (76.2)	134 (72.4)	8.89
Multiple CTO	24 (12.1)	54 (17.3)	0.11	-15.9	24 (13.0)	25 (13.5)	-1.66
New generation stent	66 (33.2)	168 (53.7)	< 0.001	-43.45	64 (34.6)	72 (38.9)	-9.16
Total stent number	1.73 (±0.87)	1.85 (±0.91)	0.13	-14.32	1.76 (±0.87)	1.76 (±0.90)	0
Total stent length	35.3 (±15.94)	38.5 (±18.90)	0.05	-20.22	35.8 (±16.16)	35.9 (±18.86)	-0.75
Minimum stent diameter	3.06 (±0.43)	2.91 (±0.42)	< 0.001	16.5	3.06 (±0.43)	3.07 (±1.60)	-2.21

Data are presented as mean ± standard deviation or n (%).

DAPT, dual antiplatelet therapy; CTO, chronic total occlusion.

The > 12-month group showed higher rate of newer generation stents, longer total stent length, and smaller minimal stent diameter compared to ≤ 12-month DAPT. The types of stents were described in S1 Table. After performing propensity-score matching for the entire population, a total of 185 pairs of matched data set were generated by 1:1 individual matching without replacement using propensity score. There were no significant differences in baseline clinical or lesion characteristics between the two groups for the propensity-matched subjects (Tables 1 and 2).

### Clinical outcomes

**Fig 3:**
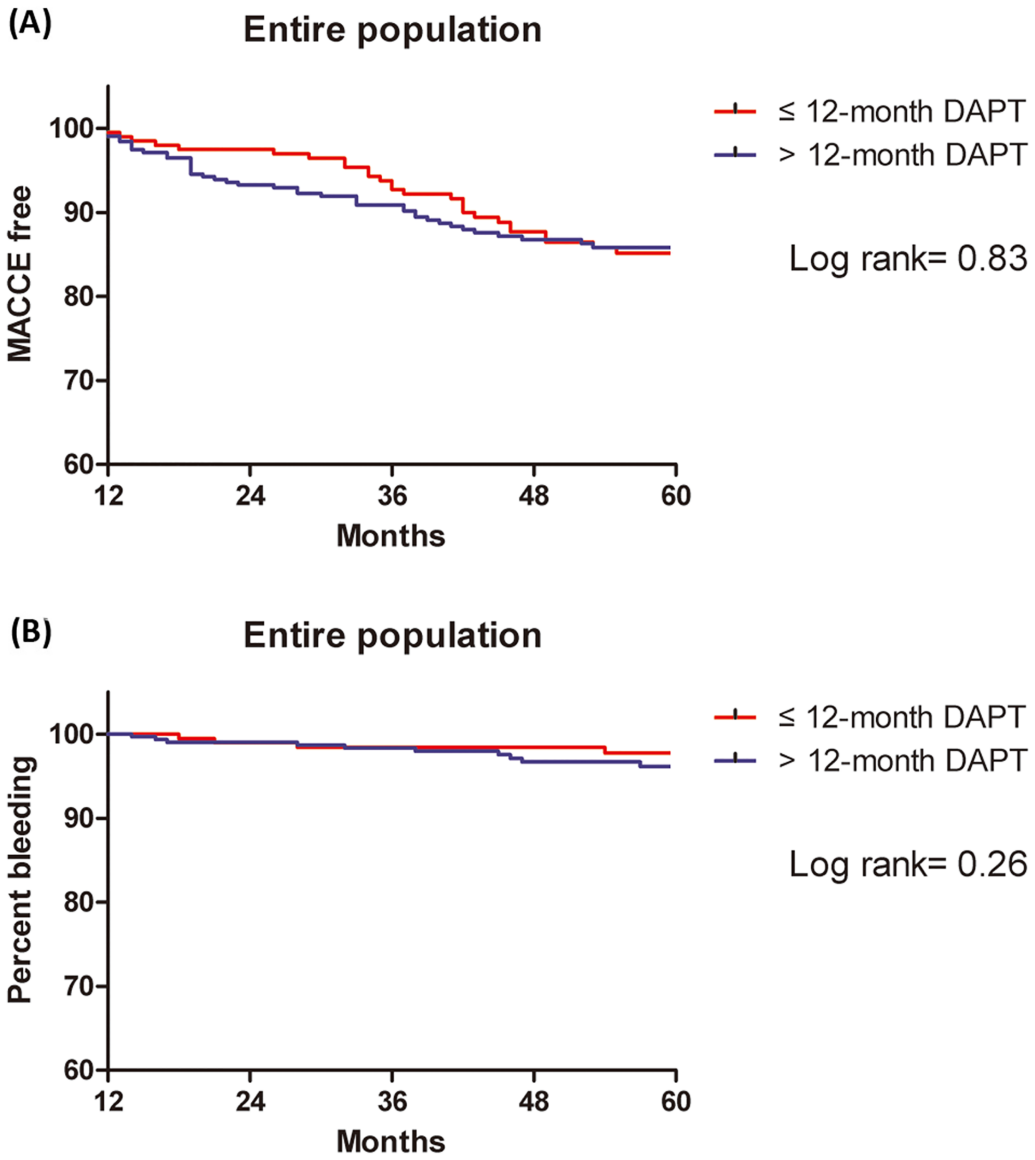
Kaplan-Meier curves for MACCE and moderate to severe bleeding between ≤ 12-month (red line) and >12-month DAPT (blue line) groups in the entire population. (A) curves for MACCE and (B) curves for moderate to severe bleeding (BARC 2, 3 or 5).

The median follow-up duration was 64 months (IQR 48 to 82) for the entire population. Table 3 shows clinical outcomes in the ≤ 12-month and > 12-month DAPT groups in the entire population. There was no significant difference between the two groups in rate of MACCE (21.6% vs. 17.6%; adjusted hazard ratio [HR] 1.27, 95% confidence interval [CI] 0.83–1.94, p = 0.26) in multivariate analysis (Table 3, Fig 3).

**Table 3:** Clinical outcomes of ≤ 12-month DAPT vs > 12-month DAPT in the crude population.

	≤ 12-month DAPT	> 12-month DAPT	Unadjusted HR (95% CI)	p-value	Multivariate analysis‡	
					Adjusted HR (95% CI)	p-value
MACCE	43 (21.6)	55 (17.6)	1.05 (0.70–1.56)	0.83	1.27 (0.83–1.94)	0.26
Stent thrombosis*	4 (2.0)	5 (1.6)	1.08 (0.29–4.03)	0.91	0.96 (0.90–7.00)	0.53
All cause death	13 (6.5)	16 (5.1)	1.18 (0.56–2.45)	0.67	1.60 (0.73–3.53)	0.24
Cardiac death	6 (3.0)	5 (1.6)	1.67 (051–5.50)	0.40	2.12 (0.60–7.56)	0.25
Myocardial infarction	3 (1.5)	10 (3.2)	0.41 (0.11–1.49)	0.18	0.54 (0.14–2.12)	0.38
Stroke	2 (1.0)	4 (1.3)	0.72 (0.13–3.92)	0.33	1.13 (0.19–6.57)	0.89
Repeat revascularizaton	29 (14.6)	36 (11.5)	1.05 (0.64–1.71)	0.72	1.21 (0.72–2.03)	0.47
BARC type 2, 3 or 5	4 (2.0)	11 (3.5)	0.52 (0.17–1.64)	0.27	0.75 (0.82–2.77)	0.75
GUSTO severe or moderate	1 (0.5)	8 (2.6)	0.17 (0.02–1.40)	0.10	0.26 (0.03–2.23)	0.22
TIMI major or minor	1 (0.5)	7 (2.2)	0.20 (0.02–1.61)	0.13	0.31 (0.04–2.67)	0.29

Data are presented as n (%).

DAPT, dual antiplatelet therapy; BARC, Bleeding Academic Research Consortium; GUSTO, Global Utilization of Streptokinase and Tissue Plasminogen Activator for Occluded Arteries; TIMI. Thrombolysis in Myocardial Infarction; CI, confidence interval; HR, hazard ratio

^‡^ Covariates include age, diabetes, hypertension, chronic kidney disease, history of PCI, history of stroke, newer generation stent, total stent length, and minimum stent diameter

* Stent thrombosis includes definite or probable stent thrombosis

**Fig 4:**
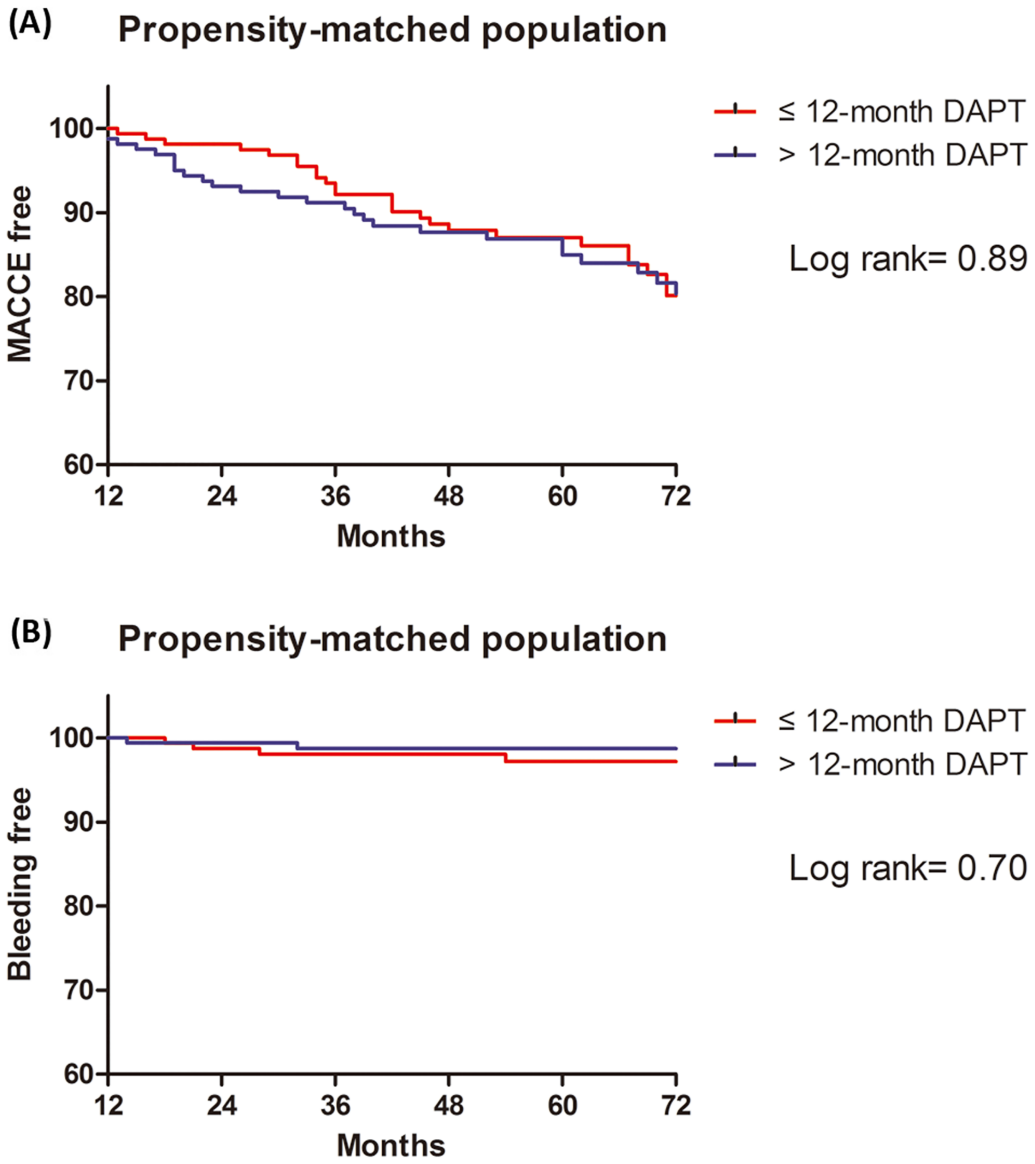
Kaplan-Meier curves for MACCE and moderate to severe bleeding between ≤ 12-month (red line) and >12-month DAPT (blue line) groups in the propensity-matched population. (A) curves for MACCE and (B) curves for moderate to severe bleeding (BARC 2, 3 or 5).

Moreover, moderate or severe bleeding according to BARC criteria (type 2, 3 or 5) (2.2% vs. 3.5%; HR, 1.14; 95% CI, 0.32–4.07, p = 0.85) was also similar between two groups. After propensity-matching, either the rate of MACCE (22.2% vs. 16.8%; HR, 1.32; 95% CI, 0.81–2.18; p = 0.26) or moderate or severe bleeding (1.6% vs. 2.2%; HR, 0.76; 95% CI, 0.17–3.44; p = 0.72) showed no differences (Table 4, Fig 4).

**Table 4:** Clinical outcomes of ≤ 12-month DAPT vs > 12-month DAPT in the propensity-matched population.

	≤ 12-month DAPT	> 12-month DAPT	Adjusted HR (95% CI)	p-value
MACCE	41 (22.2)	31 (16.8)	1.32 (0.81–2.18)	0.26
Stent thrombosis*	4 (2.2)	4 (2.2)	0.99 (0.30–3.26)	0.98
All cause death	11 (5.9)	7 (3.8)	1.58 (0.60–4.17)	0.35
Cardiac death	5 (2.7)	3 (1.6)	1.67 (0.40–7.07)	0.48
MI	3 (1.6)	6 (3.2)	0.50 (0.15–1.68)	0.30
Stroke	2 (1.1)	0		
Repeat revascularizaton	29 (15.7)	24 (13.0)	1.22 (0.69–2.13)	0.50
BARC type 2, 3 or 5	3 (1.6)	4 (2.2)	0.76 (0.17–3.44)	0.72
GUSTO severe or moderate	1 (0.5)	4 (2.2)	0.25 (0.03–2.30)	0.22
TIMI major or minor	1 (0.5)	3 (1.6)	0.34 (0.03–3.30)	0.35

Values are n (%).

DAPT, dual antiplatelet therapy; BARC, Bleeding Academic Research Consortium; GUSTO, Global Utilization of Streptokinase and Tissue Plasminogen Activator for Occluded Arteries; TIMI. Thrombolysis in Myocardial Infarction; CI, confidence interval; HR, hazard ratio

* Stent thrombosis includes definite or probable stent thrombosis

**Fig 5:**
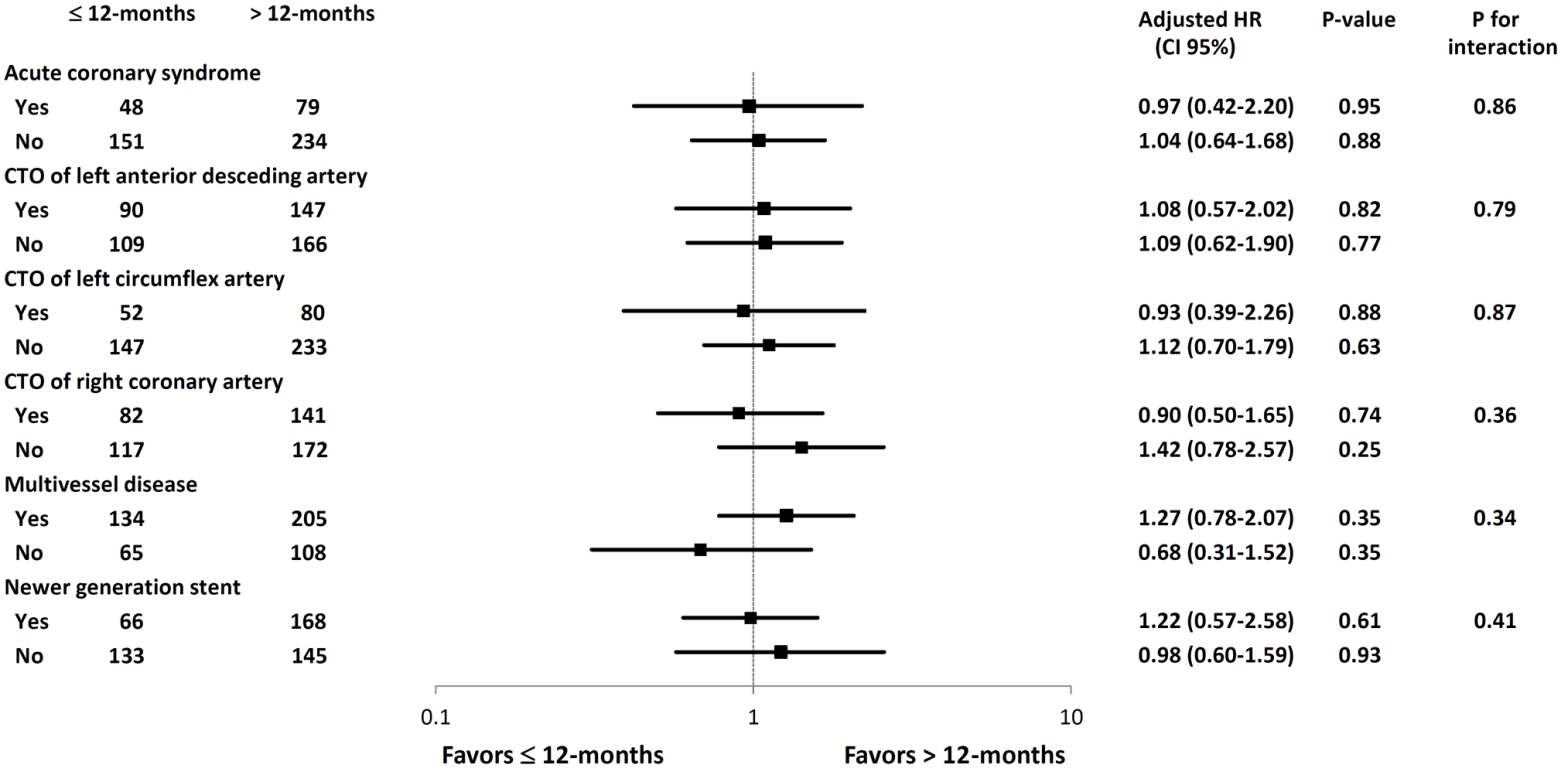
Comparison of MACCE in the subgroups. 95%CI, 95% confidence interval; HR, hazard ratio; CTO, chronic total occlusion.

There were no significant interactions between DAPT duration and baseline, lesion and procedural characteristics of interest with respect to the MACCE, as described in the hazard-ratio plots in Fig 5.

## Discussion

In the present study, we investigated clinical outcomes according to the duration of DAPT in patients treated PCI for CTO. Among patients who were event-free at 12 months, no significant differences were observed in the incidence of MACCE or moderate to severe bleeding between patients with CTO taking > 12-month DAPT and those changed to single antiplatelet therapy in 12 month.

Among patients receiving DES for non-ACS indication, current 2016 ACC/AHA guidelines and 2014 ESC/EACTS Guidelines on myocardial revascularization recommend the duration of DAPT for 6 months after DES implantation.[1, 16] Physicians tend to prefer prolonged DAPT in high-risk patients. Gilard and Morice explained that there is a possibility that only high-risk patients with conditions such as prior MI, multiple stents, left main stent, prior stent thrombosis, diabetes or diffuse coronary artery disease benefit from long- duration DAPT.[8] Generally, patients with CTO tend to be older and show higher incidences of comorbidities such as diabetes, hypertension or stroke.[9] Furthermore, coronary arteries with CTO show increased atherosclerotic burden, calcification, and longer lesions which contributes to higher SYNTAX score. Thus, it could be anticipated that patients with CTO are at higher ischemic risk and therefore benefit from longer duration of DAPT because patients treated with PCI for CTO have longer stent lengths or multiple stents.[9, 17] However, to date, limited data are available regarding the relationship between duration of DAPT and clinical outcomes in patients with CTO. The EuroCTO club reported good results after DAPT of 12 months in patients treated with sirolimus-eluting stents with non-erodible matrix and 6 months in patients treated with sirolimus-eluting stents with biodegradable polymers.[12, 18, 19] Most clinical trials of DAPT excluded patients with CTO, and even in several trials that included patients with CTO, additional subgroup analysis was not performed to investigate the clinical impact of prolonged DAPT in CTO patients.[4, 20–22] Recently published data showed that longer duration DAPT was not associated with improved clinical outcomes in the CTO patients, although other subset of complex PCI, such as longer stent length, bifurcation stenting or number of stents more than three, showed better clinical outcomes.[23] To the best of our knowledge, this is the first study performed a direct comparison of DAPT durations in patients with CTO-PCI.

Several previous studies suggest that there is no clinical benefit of longer duration DAPT.[21, 24] A recent meta-analysis argued that the risk/benefit ratio between stopping or continuing DAPT after an initially recommended period should be carefully individualized considering the trade-off between future risk of ischemia and bleeding.[25] The results of the present study are informative regarding the individualized DAPT.

In this study, we observed no differences in clinical outcomes indicating ischemic events between the ≤ 12-month and > 12-month DAPT groups. There are several possible explanations for these results. First, patients with CTO present more often with stable coronary disease than ACS.^8^ As a result, the frequency of thrombotic ischemic events, such as stent thrombosis might not differ, despite high lesion complexity and longer lesions. Patients with ST-segment elevation MI were excluded from this study, and among patients without MI, composite end points show no significant difference between prolonged DAPT and 12-months DAPT.[26] Therefore, the characteristics of patients with CTO may explain the similar rates of MACCE and stent thrombosis between groups. Second, we excluded patients with a history of PCI for more precise comparisons between groups. Study populations of previous studies that favored longer duration DAPT showed a higher incidence of prior PCI or MI than the present study.[5, 6]

The most concerning outcome of prolonged DAPT is increased bleeding risk.[27] In this study, numerically higher rates of bleeding events were observed in the > 12-month group compared to ≤ 12-month group, but the differences between the two groups were not statistically significant. Larger registry data or randomized trial is needed to evaluate clinical relevance.

Our study has several limitations. First, it was not a randomized trial: the selection of treatment was influenced by patient characteristics and patient and physician preferences. To address this limitation, we conducted propensity score-matched analysis to adjust for differences in patient characteristics. However, a strong possibility remains that other unmeasured or undocumented factors may have confounded the relationship between treatment strategies and outcomes, despite extensive adjustment for baseline risk factors. Second, the number of subjects was small as we targeted patients with successful PCI only. Third, the DAPT regimen consisted of aspirin and clopidogrel. During the study period, P2Y12 receptor inhibitors such as prasugrel or ticagrelor were not available in Korea. Fourth, the duration of DAPT is at the discretion of the physician. Finally, due to limitations of landmark analysis, there may be a possibility of omission of time-to-event distribution prior to landmark time and data-driven results. Despite these limitations, our study provides data for long-term follow-up and offers direct comparisons according to the duration of DAPT.

## Conclusion

In patients treated with PCI for CTO, the ≤ 12-month DAPT associated with similar rates of MACCE and moderate to severe bleeding compared to the > 12-month DAPT. Larger randomized controlled trials are needed to validate our results.

## Supporting information

S1 Table Types of stents. (DOCX)
