# Supplementary material for: Duration of dual antiplatelet therapy in patients treated with percutaneous coronary intervention for coronary chronic total occlusion
Source: PLoS One. 2017 May 5;12(5):e0176737. doi: 10.1371/journal.pone.0176737 (PMC5419557; doi:10.1371/journal.pone.0176737)
Supplement: S1 Table — (DOCX) [file pone.0176737.s001.docx]

| Supplementary table 1. Types of stents | |  |
| --- | --- | --- |
|  |  |  |
|  | ≤ 12-month DAPT (N=199) | > 12-month DAPT (N=313) |
| Paclitaxel-eluting stent | 44 (22.1) | 45 (14.4) |
| Sirolimus-eluting stent | 88 (44.2) | 100 (31.9) |
| Zotarolimus-eluting stent | 29 (14.6) | 62 (19.8) |
| Biolumus-eluting stent | 15 (7.5) | 26 (8.3) |
| Everlolimus-eluting stent | 23 (11.6) | 80 (25.6) |
